# Fixation artefact in an intra-operative frozen section: a potential cause of misinterpretation

**DOI:** 10.1186/1749-8090-2-45

**Published:** 2007-10-25

**Authors:** Andrew M Thomson, William A Wallace

**Affiliations:** 1 Division of Pathology, College of Medicine and Veterinary Medicine, The University of Edinburgh and Department of Pathology, Royal Infirmary of Edinburgh, Edinburgh

## Abstract

The intra-operative histological assessment of fresh tissue can provide valuable diagnostic information and guide surgical management, however, even a limited exposure to standard fixation agents can potentially compromise analysis. Defined handling strategies should exist to facilitate the receipt of all specimens, in their optimal state, by the laboratory.

## 

Sir: The intra-operative histological assessment of fresh tissue can provide valuable diagnostic information and guide surgical management [1-4]. However, the production of stained sections from frozen tissue is a technically demanding procedure which is associated with a variety of artefacts that limit interpretation and restrict subsequent clinical impact [5]. As the following example illustrates, it is important that the tissue received by the laboratory is fresh and has not been inadvertently exposed to standard fixation agents prior to analysis.

We received a small biopsy, from an incidental lung lesion found in an 81-year-old male undergoing coronary artery bypass surgery, with a request to perform an intra-operative frozen-section. The tissue had been placed in a standard specimen container to facilitate transport; as a consequence, upon arrival, it had been exposed to formalin-buffered saline for approximately 5–10 minutes. Following consultation with the surgical team, and in light of the limited period of exposure to fixative, it was decided to proceed with analysis.

The initial histology (Figure 1A) suggested a range of possible diagnoses from: a mesenchymal lesion to adipose tissue associated with a cluster of atypical epithelial cells; in contrast, cryostat analysis of a second fresh biopsy, submitted without fixation, showed features typical of squamous carcinoma (Figure 1B). This diagnosis was confirmed by the formal paraffin sections from the original specimen in which, clusters of atypical cells containing large, pleomorphic nuclei, a prominent nucleolus and eosinophilic cytoplasm were readily identified (Figure 1C). Upon review of the original sections (Figure 1A), it was clear that artefactual 'ballooning' of the neoplastic cells had occurred probably as a consequence of partial fixation and subsequent freezing of the specimen.

**Figure 1:**
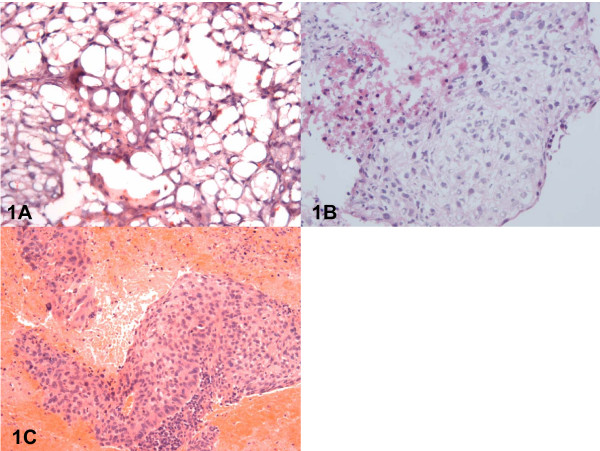
(1A): Representative cryostat section of the first biopsy (exposed, prior to freezing, to 10% neutral buffered formalin) stained with haematoxylin and eosin (magnification ×400); (1B) Representative cryostat section of the second biopsy (which had not been exposed to fixative prior to freezing) stained with haematoxylin and eosin (magnification ×400); (1C) Formal paraffin section of the first biopsy stained with haematoxylin and eosin (magnification ×200).

This case illustrates that even transient tissue fixation can potentially compromise frozen-section analysis. Defined handling strategies should exist to facilitate the receipt of all specimens, in their optimal state, by the laboratory; when these protocols are not followed, extreme care should be taken in interpreting the histology.

## Competing interests

The author(s) declare that they have no competing interests.

## Authors' contributions

All pertinent histological sections were viewed by both authors. The manuscript was drafted by AMT and reviewed and edited by WAW. Both authors read and approved the final manuscript.

## References

[B1] Shayan K, Langer JC, Smith C (2004). Reliability of intraoperative frozen sections in the management of Hirschsprung's disease. J Pediatr Surg.

[B2] Westra WH, Pritchett DD, Udelsman R (1998). Intraoperative confirmation of parathyroid tissue during parathyroid exploration: a retrospective evaluation of the frozen section. Am J Surg Pathol.

[B3] Reyes MG, Homsi MF, McDonald LW, Glick RP (1991). Imprints, smears, and frozen sections of brain tumors. Neurosurgery.

[B4] Sienk A, Allen TC, Zander DS, Cagle PT (2005). Frozen section of lung specimens. Arch Pathol Lab Med.

[B5] Desciak EB, Maloney ME (2000). Artifacts in Frozen Section Preparation. Dermatol Surg.

